# Dietary flaxseed oil rich in omega-3 suppresses severity of type 2 diabetes mellitus via anti-inflammation and modulating gut microbiota in rats

**DOI:** 10.1186/s12944-019-1167-4

**Published:** 2020-02-07

**Authors:** Lili Zhu, Liping Sha, Ke Li, Zhen Wang, Ting Wang, Yiwei Li, Ping Liu, Xiaoying Dong, Youping Dong, Xiaoxia Zhang, Hao Wang

**Affiliations:** 1 Department of Pathogenic Biology and Medical Immunology, School of Basic Medical Sciences, Ningxia Medical University, Yinchuan, 750004 Ningxia China; 2 Endocrinology Department, General Hospital of Ningxia Medical University, Yinchuan, 750004 Ningxia China; 3 Endocrinology Department, People’s Hospital of Ningxia Hui Autonomous Region, Yinchuan, 750002 Ningxia China; 4 Clinical Medical College, Ningxia Medical University, Yinchuan, 750004 Ningxia China; 5 College of Traditional Chinese Medicine, Ningxia Medical University, Yinchuan, 750004 Ningxia China

**Keywords:** Flaxseed oil, T2DM, Anti-inflammation, Gut microbiota

## Abstract

**Background:**

Type 2 diabetes mellitus (T2DM) is closely associated with hyperglycemia, abnormal lipid profiles, chronic low-grade inflammation and gut dysbiosis. Dietary intervention plays a crucial role in the control of diabetes. Flaxseed oil (FO), a plant-derived omega-3 (ω-3) polyunsaturated fatty acids (PUFAs), is rich in α-linolenic acid (ALA) which has been proved to benefit for chronic metabolic disease. However, the exact effects of dietary FO on T2DM remains largely unclear.

**Methods:**

In the present study, SD rats were randomly allocated into four groups: pair-fed (PF) with corn oil (CO) group (PF/CO); DM with CO group (DM/CO); PF with FO group (PF/FO); DM with FO group (DM/FO). A diabetic rat model was generated by a single intraperitoneal injection of streptozotocin-nicotinamide (STZ-NA). After 5 weeks of intervention, rats were euthanized and associated indications were investigated.

**Results:**

Dietary FO significantly reduced fasting blood glucose (FBG), glycated hemoglobin (GHb), blood lipid, plasma lipopolysaccharide (LPS), interleukin (IL)-1β, tumor necrosis factor (TNF)-α, IL-6, IL-17A and malondialdehyde (MDA), compared to control group, respectively. Moreover, body mass (BM) and superoxide dismutase (SOD) in DM/FO group were dramatically increased respectively, compared with those in DM/CO group. But insulin (INS) and homeostasis model assessment of insulin resistance (HOMA-IR) remained no significant difference between DM/CO group and DM/FO group. Sequencing analysis of gut microbiota showed a reduction in the relative abundance of *Firmicutes* and *Blautia*, as well as a reduction in the ratio of *Bacteroidetes-Firmicutes* in DM/FO group compared to DM/CO group. An elevation in the relative abundance of *Bacteroidetes* and *Alistipes* were detected in DM/FO group. Acetic acid, propionic acid and butyric acid belonging to short chain fatty acids (SCFAs) as gut microbiota metabolites, were dramatically increased after FO intervention. Correlation analysis revealed that the relative abundance of *Firmicutes* and *Blautia* were positively correlated with IL-1β, TNF-α, IL-6, IL-17A or LPS, respectively. Additionally, *Bacteroidetes* and *Alistipes* were negatively correlated with LPS.

**Conclusions:**

Taken together, dietary FO ameliorated T2DM via suppressing inflammation and modulating gut microbiota, which may potentially contribute to dietary control of diabetes.

## Introduction

Prevalence of T2DM presents an observably global public health problem. According to statistical data of the International Diabetes Federation, more than 450 million people suffered from diabetes worldwide in 2017, and this number was expected to hit 629 million by 2045 [1]. Thus, novel strategies for the control of T2DM are urgently needed.

Gut microbiota represents a complicated community involving bacteria in the gastrointestinal tract, which usually maintains a mutually relationship with its host. Numerous studies have demonstrated that the pathogenesis of T2DM is closely related with gut microbiota [2, 3]. It has been proven that diabetic individuals possess a high quantity of gram-negative bacteria, especially those belonging to the phylum *Proteobacteria* [4]*.* In a rat model of T2DM, the proportion of *Firmicutes* was increased, while the abundance of *Bacteroides* was decreased [5]. Regulation of gut microbiota may improve metabolism in T2DM [6].

In T2DM, gut microbiota dysbiosis promotes generation of lipopolysaccharide (LPS) triggering chronic low-degree inflammation by binding to toll-like receptor 4 (TLR-4) [7]. Excessive translocation of gut-derived LPS to the liver via portal circulation can subsequently promote the inflammatory cascade, leading to the release of inflammatory cytokines involving interleukin (IL)-1β, IL-6, IL-17A and tumor necrosis factor (TNF)-α [8]. In addition to LPS, other metabolites (such as short chain fatty acids, SCFAs) of gut microbiota have been demonstrated to target multiple pathways in intestine, liver, pancreas and peripheral blood circulation, resulting in the improvement of gut homeostasis, glycemic control, lipids profile, insulin resistance and inflammation [9]. Patients with diabetes exhibited lower contents of SCFAs and the proportion of SCFAs-producing gut bacterial communities including *Bifidobacterium*, *Bacteroidetes* and *Lactobacillus* [10]. At present, SCFAs have been recognized as potential mediators involved in intestinal immune function.

Over the past few decades, human’s lifestyle has changed greatly, with increasing animal food consumption and dietary fat intake. Incidence of T2DM was positively related to these unhealthy lifestyles, indicating that dietary factors play a crucial role in the onset and progression of T2DM [11]. One of the most common dietary approaches against T2DM is the increase in the consumption of omega-3 (ω-3) polyunsaturated fatty acids (PUFAs) [12, 13]. A survey has suggested that the low incidence of diabetes in Greenland Eskimos may partly due to high consumption of fish oil which is rich in ω-3 PUFAs, especially eicosapentaenoic acid (EPA) and docosahexaenoic acid (DHA) [14]. Flaxseed oil (FO), a plant source of ω-3 PUFAs, has shown to benefit to chronic metabolic, such as diabetes [15]. The advantage of FO over fish oil is that the food sources are less expensive and are free of heavy metal contamination. In addition, FO has been demonstrated to suppress the production of TNF-α, IL-6 and IL-1β [16, 17]. Accumulating studies have shown that FO protects against inflammation [8] and oxidative stress [18] in metabolic disease models. However, the relationships among dietary FO, chronic inflammation and gut microbiota in diabetic rats still remains largely unclear.

The present study aimed to assess the effects of dietary FO and mechanisms related to inflammation and gut microbiota in diabetic rats. Our study may contribute to further understanding of the complicated interaction between diet, the gut microbiota and inflammation of T2DM.

## Materials and methods

### Animals

Male Sprague-Dawley (SD) rats (200-250 g) were purchased from Central Animal House of NingXia Medical University. Rats were conditioned in standard polypropylene cages (4 rats/cage) with controlled temperature (22 ± 2 °C), humidity (40–70%) and 12 h light/12 h dark-cycle for 1 week before the experiment. All animal experiments were approved by Ethics Committee of the General Hospital of Ningxia Medical University (No. 2016–232). During the whole experimental period, animals were fed with a balanced commercial diet and water ad libitum. All diets for rats feeding were purchased from TROPHIC Animal Feed High-tech Co., Ltd., Nantong, China.

### Diabetic rats

The rats were fasted 12 h before the streptozotocin (STZ)-nicotinamide (NA) injection. T2DM was induced by the intraperitoneal (i.p) administration of a single dose (65 mg/kg BM) of STZ freshly dissolved in 0.1 M citrate buffer (pH = 4.5), 15 min after i.p administration of 110 mg/kg BM of NA dissolved in normal saline. Animals were allowed to drink 20% glucose solution overnight to overcome drug-induced hypoglycemia. Rats in the pair-fed (PF) group were i.p injected with an equal volume of citrate buffer and normal saline. Hyperglycemia was confirmed according to the levels of fasting blood glucose (FBG), which was determined in day 3 and 7 after injection. Rats with FBG levels above 13.9 mmol/L were considered to T2DM in the experiment [19], followed by feeding various types of diets.

### Experimental design

Rats were randomly divided into four groups (8 rats/group): (a) PF with corn oil (CO) group (PF/CO), PF rats were fed 10% w/w CO diet as CO control; (b) DM with CO group (DM/CO), DM rats were fed 10% w/w CO diet; (c) PF with flaxseed oil (FO) group (PF/FO), PF rats were fed 10% w/w FO diet as FO control; (d) DM with FO group (DM/FO), DM rats were fed 10% w/w FO diet. The standard rodent chow pellets were powdered and mixed with FO and re-pelleted so as to contain either 10% w/w. Animals from PF/FO group and DM/FO group received 10% w/w FO diet. As control, PF/CO group and DM/CO group received 10% w/w CO diet. The fatty acid composition of dietary fats are shown in (Additional file 1: Table S1). Feeds were prepared every week and packed sealed bags in quantities sufficient for 1 day’s feed. Feeds in the plastic bags were flushed with nitrogen, sealed, and stored at − 20 °C. Feeds which were not consumed by animals were discarded daily. During the experimental period, body mass (BM) and FBG were determined weekly to reflect the alteration of basic indicators. After 5 weeks of feeding, rats were euthanized by 4% sodium pentobarbital and associated indications were investigated. Blood samples were respectively collected with or without ethylene diamine tetraacetic acid (EDTA) and centrifuged (700×g for 10 min) to obtain plasma or serum samples. Serum samples were used to detect oxidative markers and plasma samples were used to determine the remaining chemical indicators, both of which were stored in − 80 °C before further analysis.

### Plasma biochemical tests

At the end of the experiment, glycated hemoglobin (GHb) was measured using the Glycosylated Hemoglobin (GHb) enzyme linked immunosorbent assay (ELISA) Kit (Wuhan ColorfulGene Biological Technology Co., Ltd., Wuhan, China). Plasma insulin concentration (INS) was analyzed using commercial enzyme-linked immunoassay kit (Qiaoyi Biology, Anhui, China). Homeostasis model assessment of insulin resistance (HOMA-IR) was calculated as [glucose (mmol/L) × insulin (mU/L)]/22.5. Total cholesterol (TC), triglyceride (TG), low density lipoprotein (LDL) and high density lipoprotein (HDL) levels in each group were respectively determined using AU400 automatic biochemical analyzer (Olympus, Japan).

### Oxidative indicators

Malondialdehyde (MDA) assay kits and total superoxide dismutase (SOD) activity in each group were respectively determined with thiobarbituric acid (TBA) and hydroxylamine method according to the manufacturer’s instructions (Nanjing Jiancheng Bioengineering Ins., Nanjing, China).

### Determination of plasma LPS

Plasma LPS levels of each group were determined using limulus amebocyte lysate kit (Xiamen Bioendo Technologky Co. Ltd., Xiamen, China). Briefly, 50 μL of plasma was dispensed to each well in a 96-well plate. At the initial time point, 50 μL/well of the limulus amebocyte lysate reagent was added respectively. The plate was incubated at 37 °C for 30 min. Then 100 μL of chromogenic substrate warmed to 37 °C was added to each well. Incubation was extended for an additional 6 min at 37 °C. The reaction was stopped by adding 100 μL of a 25% solution of glacial acetic acid. Optical density at 545 nm was measured with a microplate reader (Thermo Scientific, USA).

### ELISA assays

Plasma inflammatory cytokines including IL-1β, TNF-α, IL-6, IL-17A and IL-10 were measured by ELISA kits according to the manufacturer’s instructions (Shanghai Jianglai Biotech, Shanghai, China). Optical density was measured at 450 nm within 15 min, using an automated microplate reader (Thermo Fisher Scientific, USA).

### Gut microbiota analysis

The fecal microbial 16S rRNA high throughput sequencing and analysis were performed according to previous studies [3]. After treatment, 5 rats were randomly selected from each group and transferred to fresh sterilized cages. The feces of each rat were respectively collected and immediately stored at − 80 °C for subsequent DNA extraction.

Total genome DNA from samples was extracted using cetyltrimethylammonium bromide (CTAB) method as previously described [20], which was monitored by electrophoresis on 1% agarose gels (Additional file 2: Figure S1). According to the concentration, DNA was diluted to 1 ng/μL using sterile water.

The hypervariable V3 to V4 regions of the 16S rRNA gene were amplified with barcoded primers: 341F 5′- CCTAYGGGRBGCASCAG-3′ and 806R 5′- GGACTACNNGGGTATCTAAT-3′. All PCR reactions were carried out in 30 μL reactions with 15 μL of Phusion® High-Fidelity PCR Master Mix (New England Biolabs), 0.2 μM of forward and reverse primers, and 10 ng template DNA. Thermal cycling consisted of initial denaturation at 98 °C for 1 min, followed by 30 cycles of denaturation at 98 °C for 10 s, annealing at 50 °C for 30 s, and elongation at 72 °C for 30 s, finally 72 °C for 5 min. Same volume of 1 × loading buffer (contained SYB green) was mixed with PCR products and operated electrophoresis on 2% agarose gel for detection. Then, PCR products were purified with GeneJET TM Gel Extraction Kit (Thermo Scientific, USA). Sequencing libraries were generated using Ion Plus Fragment Library Kit 48 rxns (Thermo Scientific, USA) following manufacturer’s recommendations. The library quality was assessed on the Qubit@ 2.0 Fluorometer (Thermo Scientific, USA). At last, the library was sequenced on an Ion S5 TM XL platform to generate 400–600 bp single-end reads. The library was sequenced on an Illumina HiSeq 2500 platform by Beijing Nuo He Zhi Yuan Technology Co., Ltd., China.

### Fecal SCFAs quantification by gas chromatography-mass spectrometer (GC-MS)

Quantification analysis of fecal SCFAs was performed using an Agilent 7890A gas chromatography coupled with an Agilent 5975C mass spectrometric detector (Agilent Technologies, USA) equipped with an HP-5MS column (0.25 × 30 mm, 0.25 μm particlesize) (Suzhou Bionovogene Co., Ltd) as described previously [21]. Helium was used as a carrier gas at a constant flow rate of 1 mL/min. The initial oven temperature was held at 60 °C for 5 min, ramped to 250 °C at a rate of 10 °C/min, and finally held at this temperature for 5 min. The temperatures of the front inlet, transfer line and electron impact (EI) ion source were set as 280, 250 and 230 °C, respectively. Data handing was performed with an Agilent’s MSD ChemStation (E.02.00.493, Agilent Technologies, Inc., USA).

### Statistical analysis

The data are expressed as the mean ± SEM. When the data were Gaussian distribution and variances were equal, differences among the groups were analyzed using one-way ANOVA followed by Tukey’s post hoc test. Otherwise, the Kruskal-Wallis test and followed Dunn’s post hoctesting were applied. Spearman’s correlation analysis was performed to identify the correlations between microbiota and inflammatory indicators. The statistically significance level was considered as *P* < 0.05 (Prism version 5.0 for Windows; GraphPad Software, San Diego, CA, USA).

## Results

### Routine parameters of rats in diverse groups

#### Body mass (BM)

**Fig. 1:**
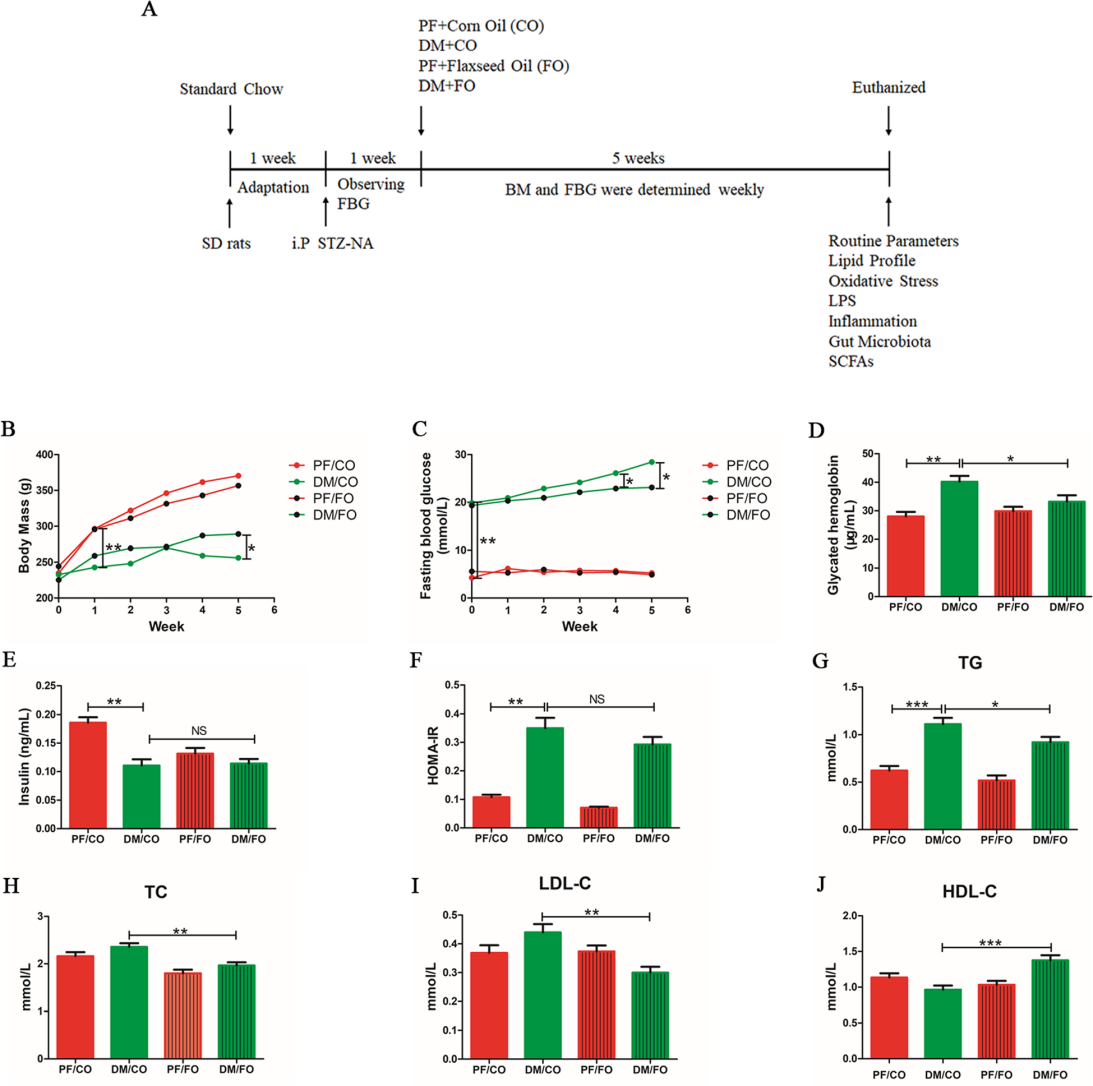
Effects of dietary flaxseed oil treatment on glucolipid metabolism in diverse groups. **a** Schematic diagram of the study. SD rats with 200-250 g were conditioned for 1 week. Then T2DM model (FBG > 13.9 mmol/L) were successfully induced by the intraperitoneal (i.p) administration of STZ-NA within another 1 week. For a subsequent period of 5 weeks, rats in diverse groups were respectively administrated with corn oil (CO) and flaxseed oil (FO) in control group (PF) or T2DM model group (DM). Body mass (BM) and FBG for each group were determined weekly. After 5 weeks of intervention, rats were euthanized and associated indications were investigated. **b** Body mass (BM); **c** Fasting blood glucose (FBG); **d** Glycated hemoglobin (GHb); **e** Insulin (INS); **f** Homeostasis model assessment of insulin resistance (HOMA-IR); **g** TG: Triglyceride; **h** TC: Total cholesterol; **i** LDL-C: Low density lipoprotein cholesterol; **j** HDL-C: High density lipoprotein cholesterol. Data was presented as mean ± SEM (*n* = 6). Data was *P < 0.05, ** *P* < 0.001, NS not significant (one-way ANOVA followed by Tukey’s post hoc test)

There was no significant difference in initial BM among diverse groups. We found that BM in DM/CO group were significantly decreased compared to PF/CO group at week 1 (*P*<0.001, Fig. 1b). However, after 5 weeks of administration, BM in DM/FO group were notably increased compared with that in DM/CO group (*P*<0.05, Fig. 1b). These results demonstrated that dietary FO can maintain the BM in T2DM.

#### Fasting blood glucose (FBG) and Glycated hemoglobin (GHb)

FBG levels were significantly increased in diabetic rats compared to control rats (Fig. 1c). Intriguingly, the levels of FBG in DM/FO group were dramatically decreased compared with that in DM/CO group at week 4 and 5 (*P*<0.05, Fig. 1c). There was no significant difference of FBG levels between PF/CO group and PF/FO group (Fig. 1c). Similarly, GHb in DM/CO group was higher than that in PF/CO group. However, the levels of GHb in DM/FO group were lower than that in DM/CO group (*P*<0.05, Fig. 1d).

#### Insulin (INS) and homeostasis model assessment of insulin resistance (HOMA-IR)

In addition, plasma INS (*P*<0.001, Fig. 1e) level were notably decrease in DM/CO group compared to PF/CO group. In DM/CO group, HOMA-IR (*P*<0.001, Fig. 1f) significantly increase compared to PF/CO group. But INS and HOMA-IR remained no significant difference (*P*>0.05) between DM/CO group and DM/FO group after the administration of dietary FO.

### Plasma lipid levels

To assess the effects of dietary FO on lipid metabolism in rats, the plasma triglyceride (TG), total cholesterol (TC), low density lipoprotein cholesterol (LDL-C) and high density lipoprotein cholesterol (HDL-C) levels were investigated (Fig. 1g-j). The results showed that concentrations of plasma TC (Fig. 1h), TG (Fig. 1g) and LDL (Fig. 1i) in DM/FO group were effectively decreased, whereas HDL levels were increased, compared to DM/CO group (*P*<0.001, Fig. 1j).

### Serum SOD activity and MDA activity

**Fig. 2:**
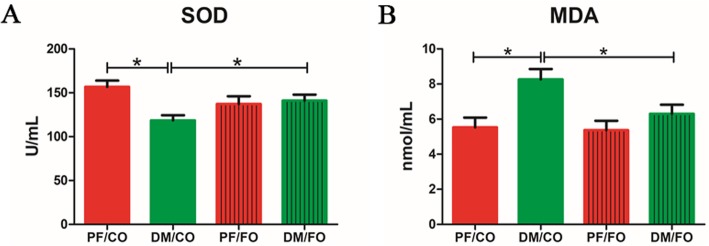
Serum oxidative marker levels in the four groups of rats. **a** superoxide dismutase (SOD); **b** malondialdehyde (MDA). * *P* < 0.05

To investigate the effects of dietary FO on oxidative stress in diabetic rats, we examined the levels of serum SOD and MDA (Fig. 2). In DM/CO group, SOD level in serum significantly reduced compared to PF/CO group. Feeding of diabetic rats with FO significantly increased SOD levels compared to the diabetic control group (*P*<0.05, Fig. 2a). However, serum MDA was significantly decreased in the supplementation with dietary FO in diabetes (*P*<0.05, Fig. 2b).

### Dietary FO reduced the plasma LPS levels

**Fig. 3:**
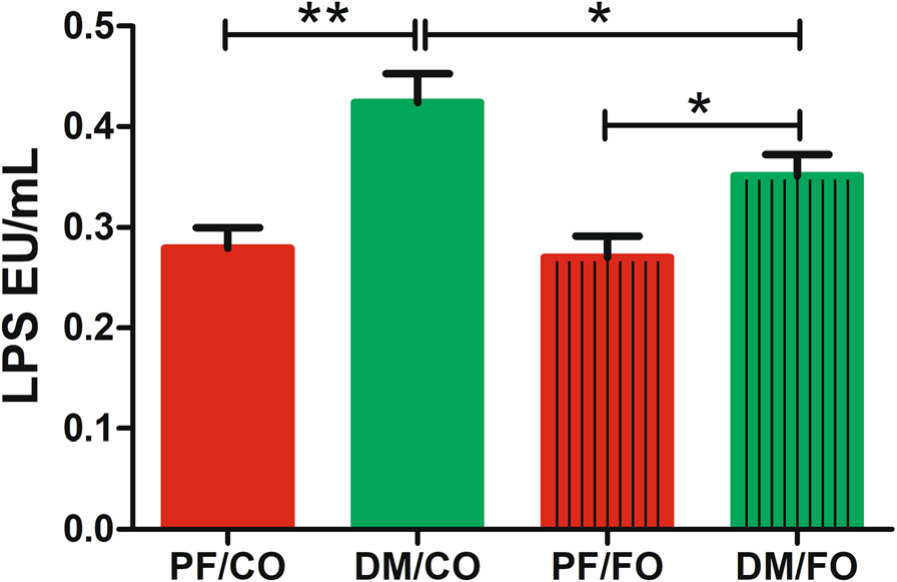
Effects of dietary flaxseed oil treatment on plasma lipopolysaccharide (LPS) levels in T2DM. * *P* < 0.05, ** *P* < 0.001

Plasma levels of LPS in DM/FO group were significantly decreased compared to DM/CO group (*P*<0.05), but still higher than that in PF/CO group or PF/FO group (Fig. 3), demonstrating that dietary FO possessed the ability to attenuate LPS generation from gram-negative pathogenic bacteria.

### Dietary FO altered plasma inflammatory cytokine levels in T2DM

**Fig. 4:**
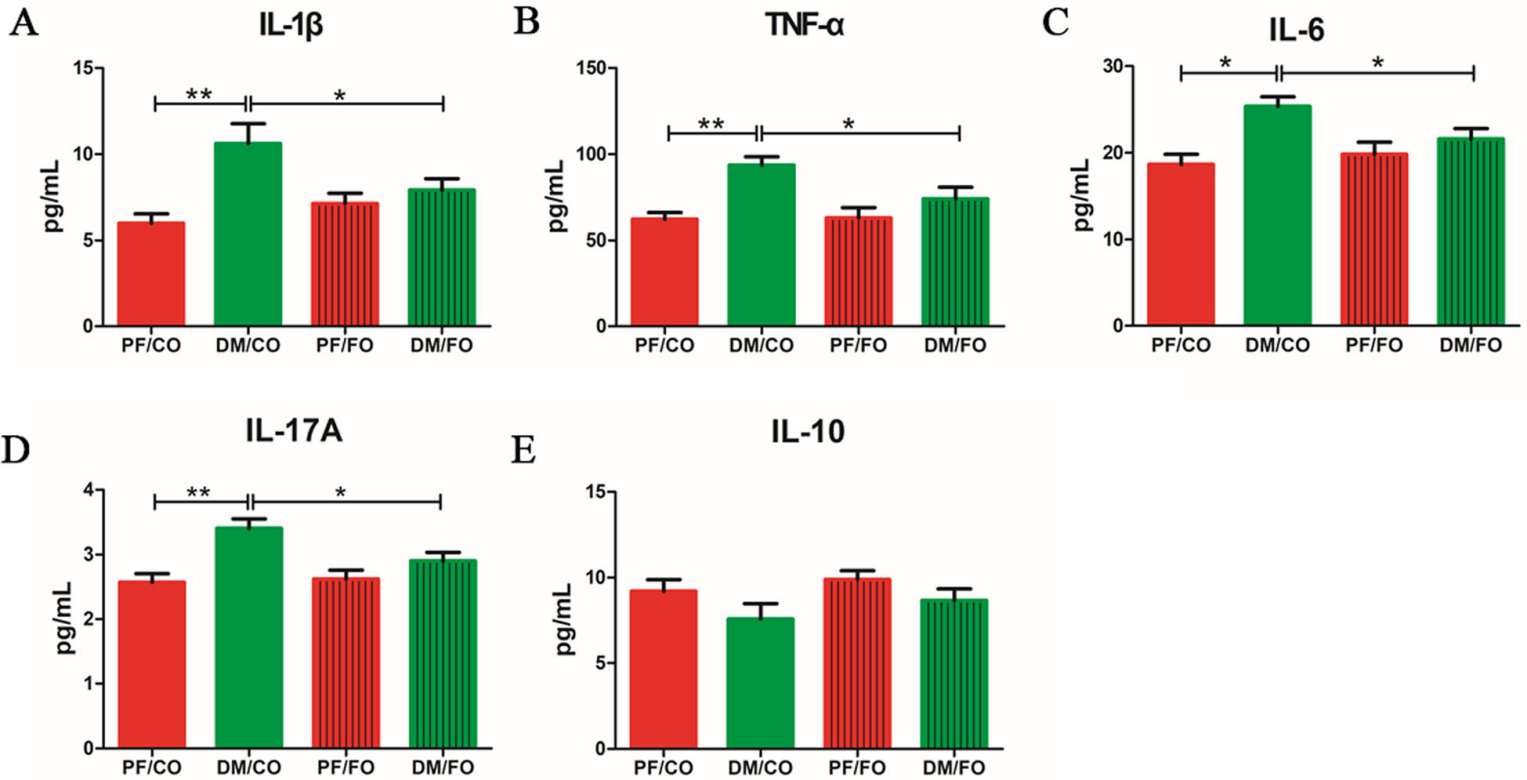
Plasma of rats from diverse groups were collected respectively for detection of IL-1β (**a**), TNF-α (**b**), IL-6 (**c**) and IL-17A (**d**) and IL-10 (**e**) concentrations using ELISA kit. * *P* < 0.05, ** *P* < 0.001

In attempt to investigate the effects of dietary FO on inflammation in diabetic rats, the levels of inflammatory cytokines including TNF-α, IL-1β, IL-6, IL-17A and IL-10 were measured, respectively (Fig. 4a-e). Our results showed that the levels of IL-1β (Fig. 4a), TNF-α (Fig. 4b), IL-6 (Fig. 4c) and IL-17A (Fig. 4d) in DM/CO group were significantly increased compared to PF/CO group, respectively. However, dietary FO administration remarkably decreased the contents of IL-1β (*P*<0.05) and TNF-α (*P*<0.05) in plasma, compared with that in DM/CO group. Similarly, plasma IL-6 (*P*<0.05) and IL-17A (*P*<0.05) levels in DM/FO were also significantly reduced in comparison with those cytokines in DM/CO group. It showed no significant difference in plasma IL-10 levels between DM/CO group and DM/FO group (*P*>0.05, Fig. 4e). There results indicated that dietary FO treatment ameliorated the level of inflammation in rats with T2DM.

### Correlation analysis of LPS and inflammatory cytokines

We analyzed the correlations among LPS and a series of inflammatory cytokines. LPS was positively correlated with IL-1β, TNF-α, IL-6 and IL-17A (Additional file 3: Figure S2).

### Dietary FO modulated gut microbiota in T2DM

**Fig. 5:**
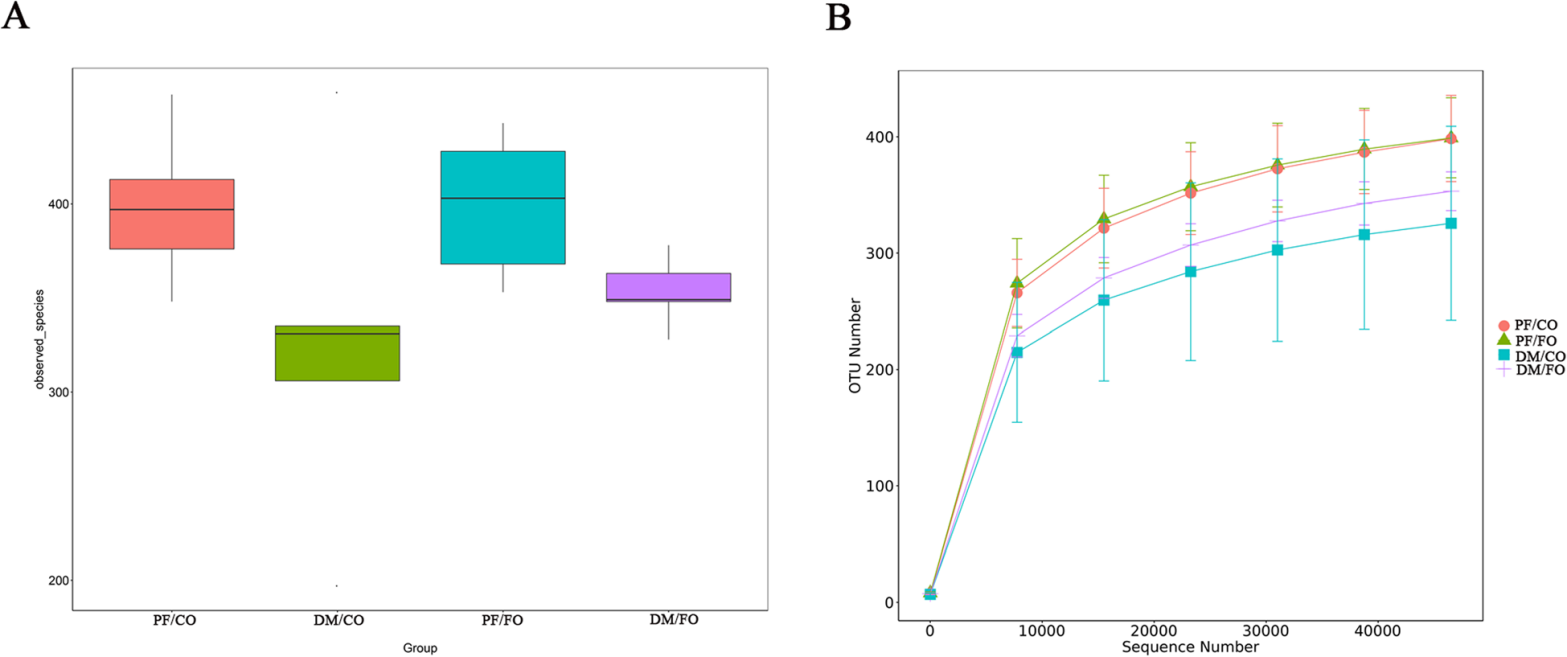
Alpha-diversity analysis showing difference in terms of abundance and diversity of gut microbiota in diverse groups. **a** Observed-species index; **b** Rarefaction curve

**Fig. 6:**
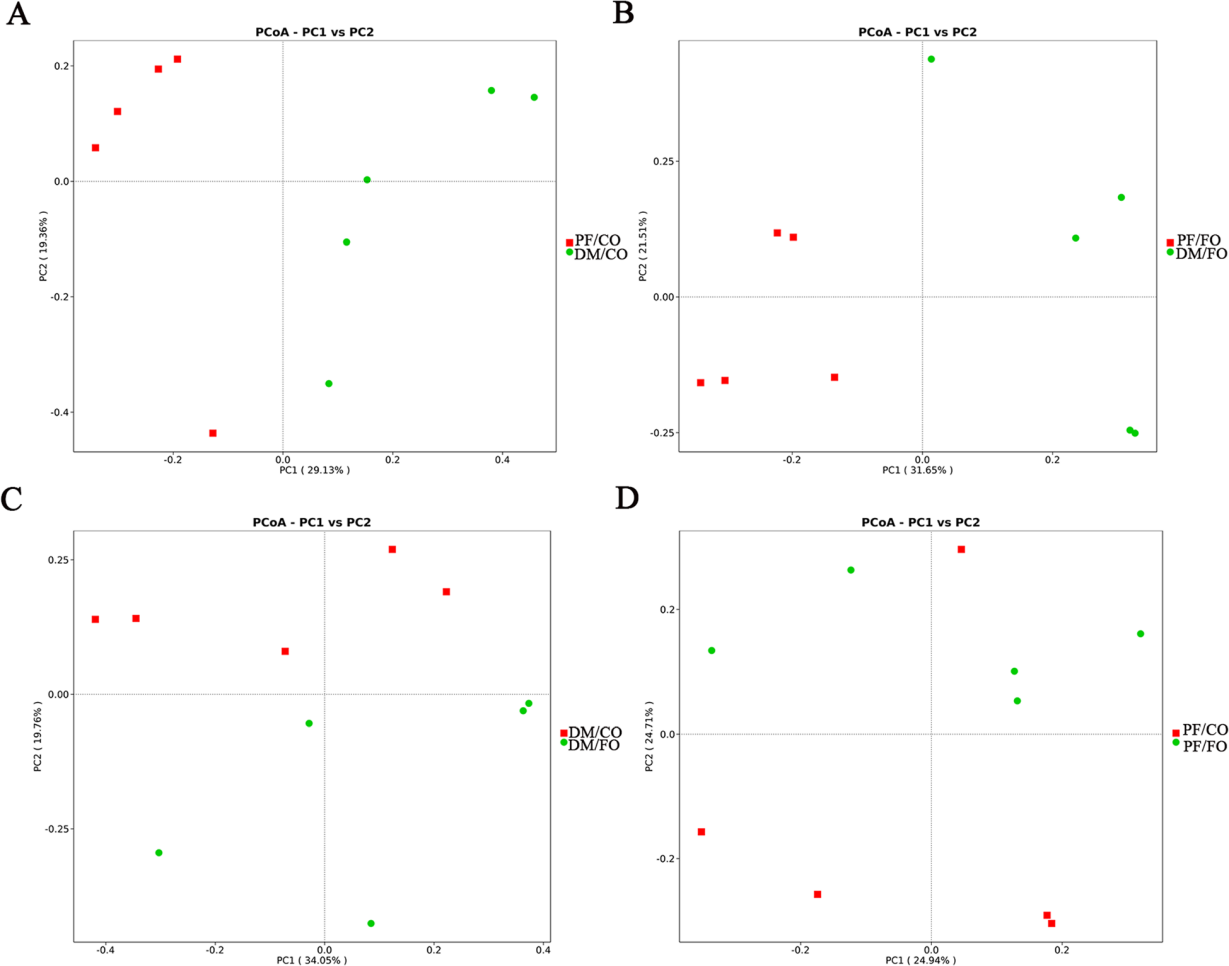
PCoA analysis showing difference in terms of species in fecal samples. Beta diversity was on unweighted UniFrac. **a** PF/CO vs. DM/CO; **b** PF/FO vs. DM/FO; **c** DM/CO vs. DM/FO; **d** PF/CO vs. PF/FO

**Fig. 7:**
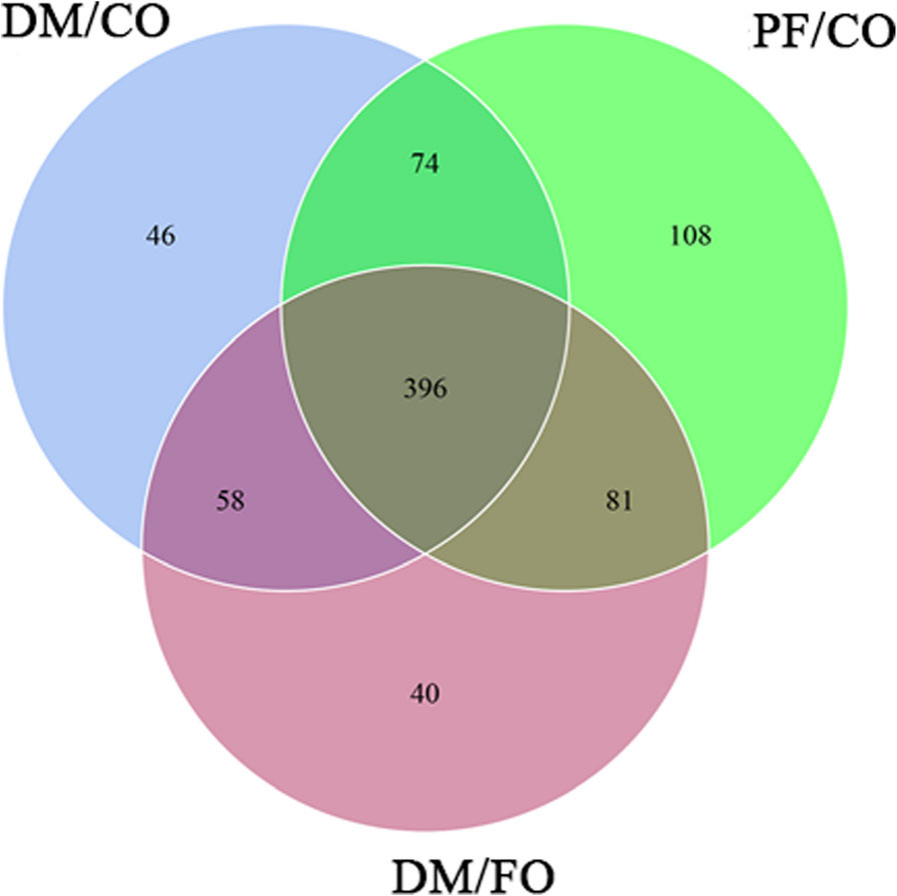
Shared OUT analysis of the different groups. Venn diagram showing the unique and shared OTUs in the different groups

Accumulating studies have been addressing the close linkage between the changes to gut microbiota structure and T2DM [3, 5, 22]. To assess whether FO administration was associated with modulation of gut microbiota composition in diabetic rats, the fecal samples were analyzed by 16S rRNA high throughput sequencing. Alpha-diversity was used to analyze the abundance and diversity of bacterial community, which was assessed by observed-species index and rarefaction curve. As shown in Fig. 5a, observed-species index analysis showed that the abundance and diversity of gut microbiota in DM/CO group were the lowest in four groups, but the abundance and diversity of gut microbiota were altered after dietary FO treatment. However, the difference showed no significance (*p* > 0.05) by Tukey’s post hoc test and Wilcoxon rank sum test. Rarefaction curves which were adopted to evaluate the rationality of the sequencing data (Fig. 5b), tended to be flat when the number of sequence increased to 10,000, showing that the amount of sequencing data were reasonable. Beta-diversity for analyzing the overall composition of bacterial community was assessed by principal coordinates analysis (PCoA) (Fig. 6) and nonmetric multidimensional scaling (NMDS). PCoA results showed a significant difference of species in fecal samples among the PF/CO group and DM/FO group compared to DM/CO group respectively (Fig. 7), indicating that dietary FO can alter gut microbiota of diabetic rats. In addition, NMDS analysis owned similar results (Additional file 4: Figure S3).

As shown above, to further explore the diversity of alpha-diversity and beta-diversity in composition of gut microbiota, the species distribution of gut microbiota from PF/CO, DM/CO and DM/FO groups were compared by operational taxonomic unit (OUT) analysis. As shown in venn diagram (Fig. 7), we found that there were 396 species shared among PF/CO, DM/CO and DM/FO groups. Intriguingly, 108 species were found in PF/CO group, 46 species in DM/CO group and 40 OTUs in DM/FO group.

**Fig. 8:**
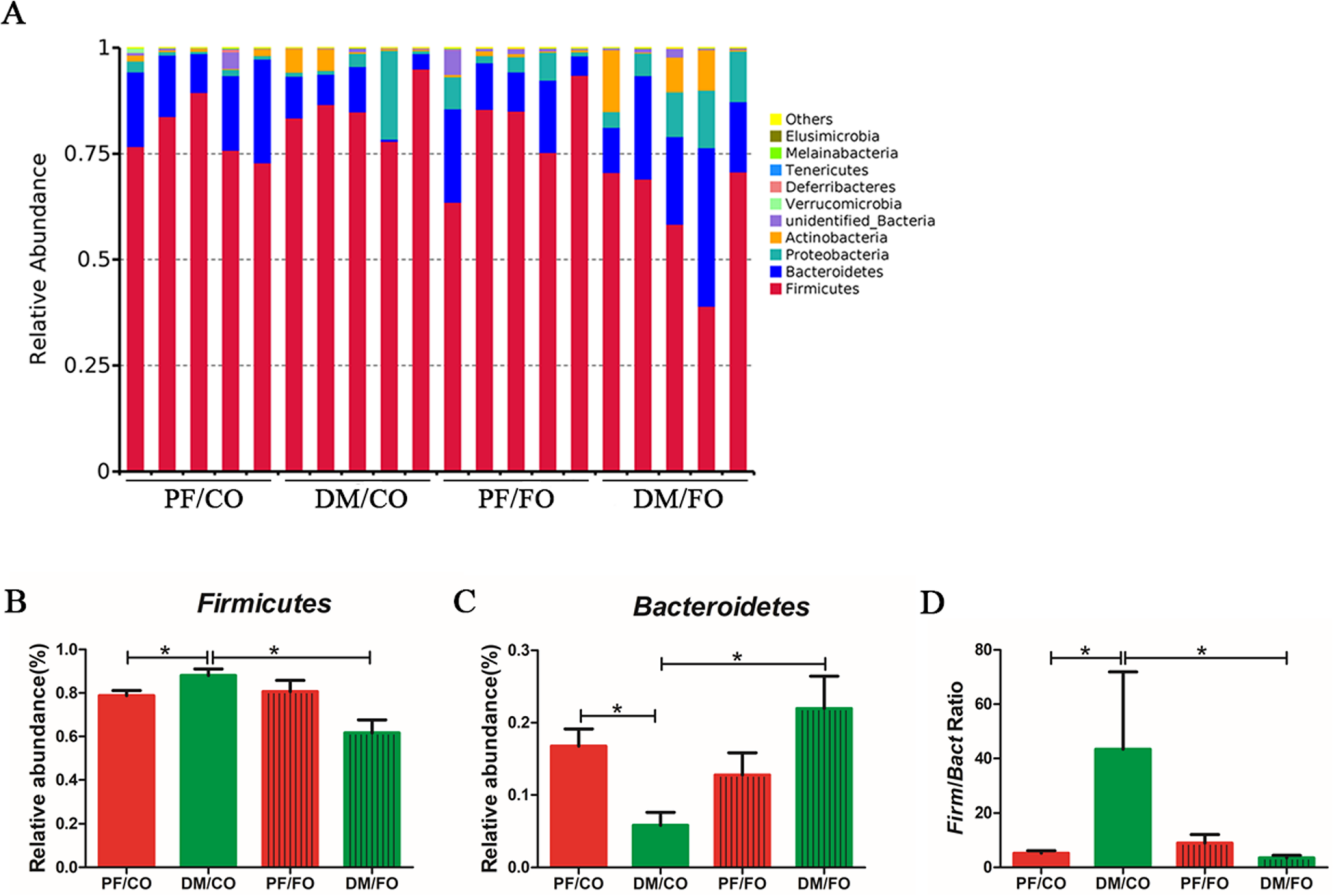
Relative abundance of microbial species at the phylum level in diverse groups of rat feces. **a** The relative abundance of microbial species at the phylum level in the feces of rats; **b***Firmicutes*; **c***Bacteroidetes*; **d** the ratio of *Bacteroidetes*-*Firmicutes*. * *P* < 0.05

After 5 weeks of FO intervention, at phylum level, we found that *Firmicutes* and *Bacteroidetes* constituted two dominant phyla in each group (Fig. 8a). The proportion of *Firmicutes* was significantly elevated in DM/CO group compared to PF/CO group, which was reversed by dietary FO (Fig. 8b). The relative abundance of *Bacteroidetes* was significantly reduced in DM/CO group than that in PF/CO group (P<0.05, Fig. 8c) or DM/FO group (*P*<0.05, Fig. 8c). In addition, our results also indicated that DM rats had a higher ratio of *Firmicutes*-*Bacteroidetes*, whereas FO intervention reduced the abnormal ratio (*P*<0.05, Fig. 8d). Taken together, our data revealed that under experimental conditions, FO supplementation had a major effect on *Firmicutes* and *Bacteroidetes.*

**Fig. 9:**
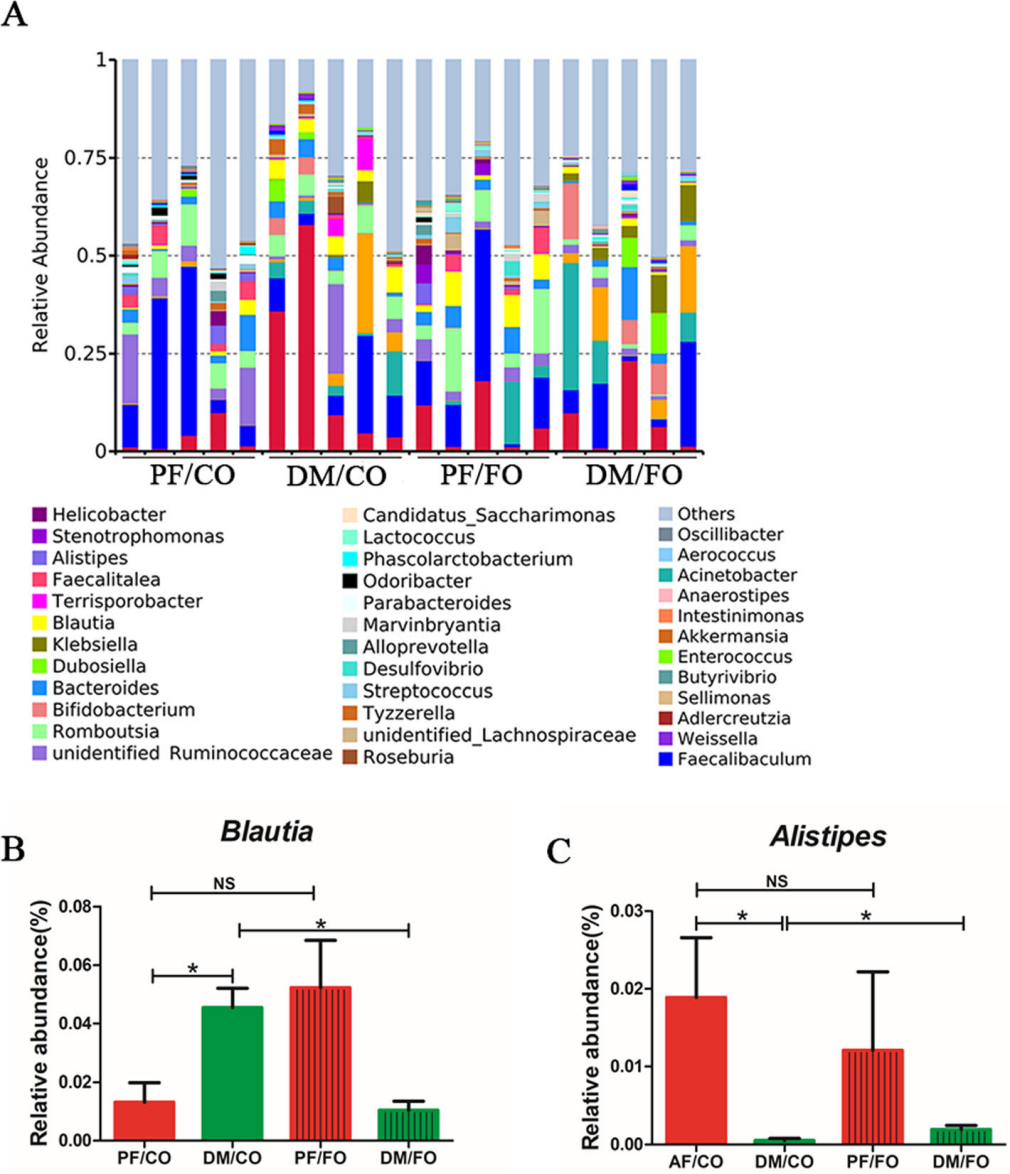
Relative abundance of microbial species at the genus level in the feces. **a** The relative abundance of microbial species at the genus level in the feces of rats; **b***Blautia*; **c***Alistipes*; * *P* < 0.05, NS not significant

To further understand the composition of gut microbiota in T2DM, we analyzed top 40 taxa of genus level in diverse groups (Fig. 9). We found that the proportion of *Blautia* in DM/CO group was higher than that in PF/CO group, whereas FO intervention obviously reduced the relative abundance (*P*<0.05, Fig. 9b). In contrast, the proportion of *Alistipes* was decreased in DM/CO group, but FO treatment sharply elevated the relative abundance of *Alistipes* in diabetic rats (*P*<0.05, Fig. 9c). Collectively, these results indicated that dietary FO obviously altered the initial proportion of OTUs at genus level, mainly including *Blautia* and *Alistipes*.

### Gut microbiota was correlated with inflammatory indicators

**Fig. 10:**
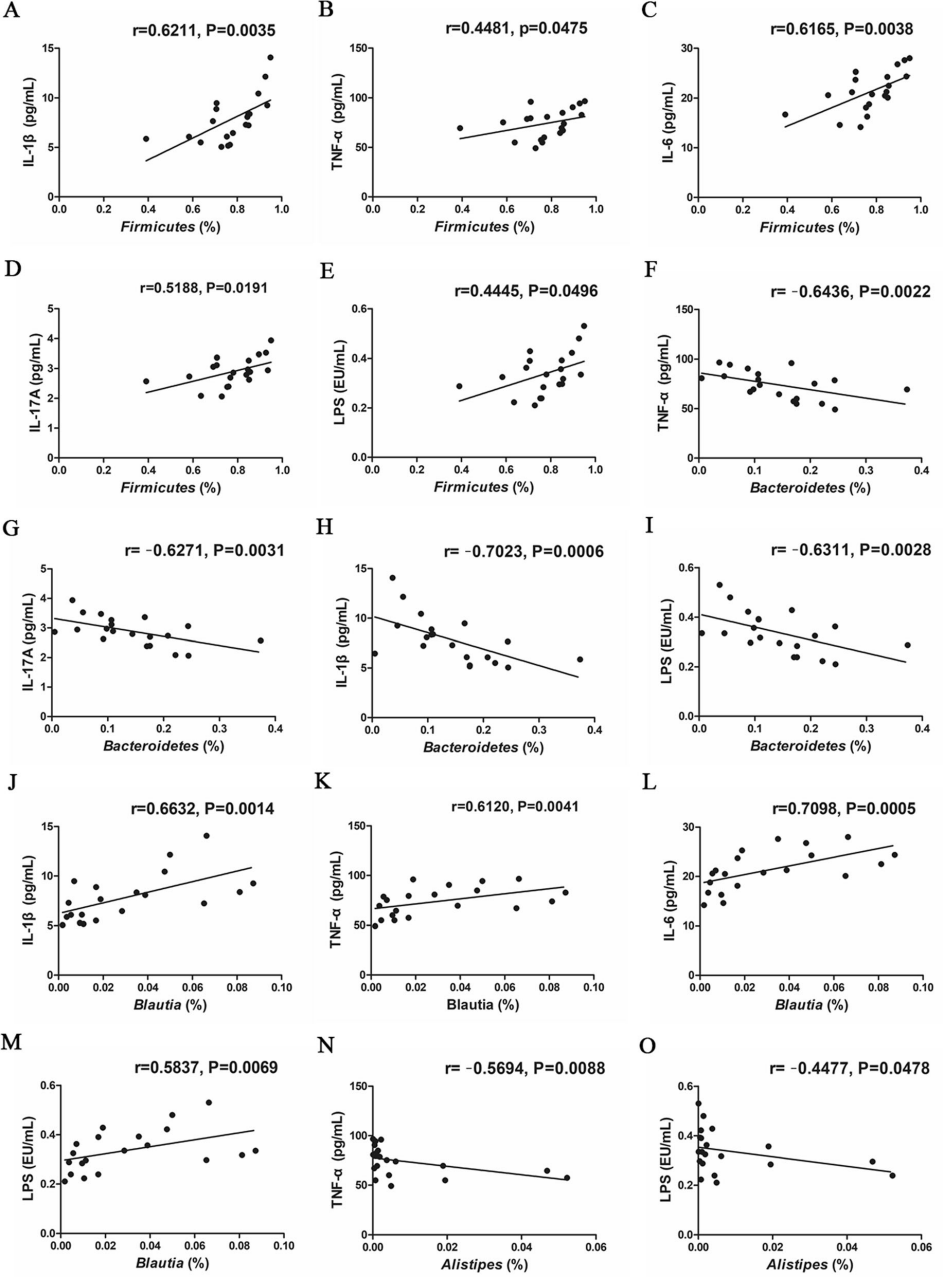
Correlations between inflammatory cytokine and bacterial abundance at the phylum level or genus in the feces of rats. **a**-**e***Firmicutes* and IL-1β, TNF-α, IL-6, IL-17A, LPS. **f**-**i***Bacteroidetes* and TNF-α, IL-17A, IL-1β, LPS. **j**-**m***Blautia* and IL-1β, TNF-α, IL-6, LPS. **n**-**o***Alistipes* and TNF-α, LPS, respectively

Correlations between a series of inflammatory indicators and the proportions of the above differential bacteria were analyzed at phylum and genus levels, respectively (Fig. 10). Our results showed that the relative abundance of *Firmicutes* was positively correlated with the levels of IL-1β (*P* = 0.0035, Fig. 10a), TNF-α (*P* = 0.0475, Fig. 10b), IL-6 (*P* = 0.0038, Fig. 10c), IL-17A (*P* = 0.0191, Fig. 10d) and LPS (*P* = 0.0496, Fig. 10e), respectively. Moreover, *Bacteroidetes* abundance was negatively correlated with IL-1β (*P* = 0.0006, Fig. 10h), TNF-α (*P* = 0.0022, Fig. 10f), IL-17A (*P* = 0.0031, Fig. 10g) and LPS (*P* = 0.0028, Fig. 10i), respectively. At genus level, the *Blautia* abundance showed a positive association with IL-1β (*P* = 0.0014, Fig. 10j), TNF-α (*P* = 0.0041, Fig. 10k), IL-6 (*P* = 0.0005, Fig. 10l) and LPS (*P* = 0.0069, Fig. 10m). *Alistipes* abundance was negatively correlated with TNF-α (*P* = 0.0088, Fig. 10n) and LPS (*P* = 0.0478, Fig. 10o). These results indicated that gut microbiota and inflammatory indicators interfered and closely correlated.

### FO increased the SCFAs in fecal intestinal metabolites

**Fig. 11:**
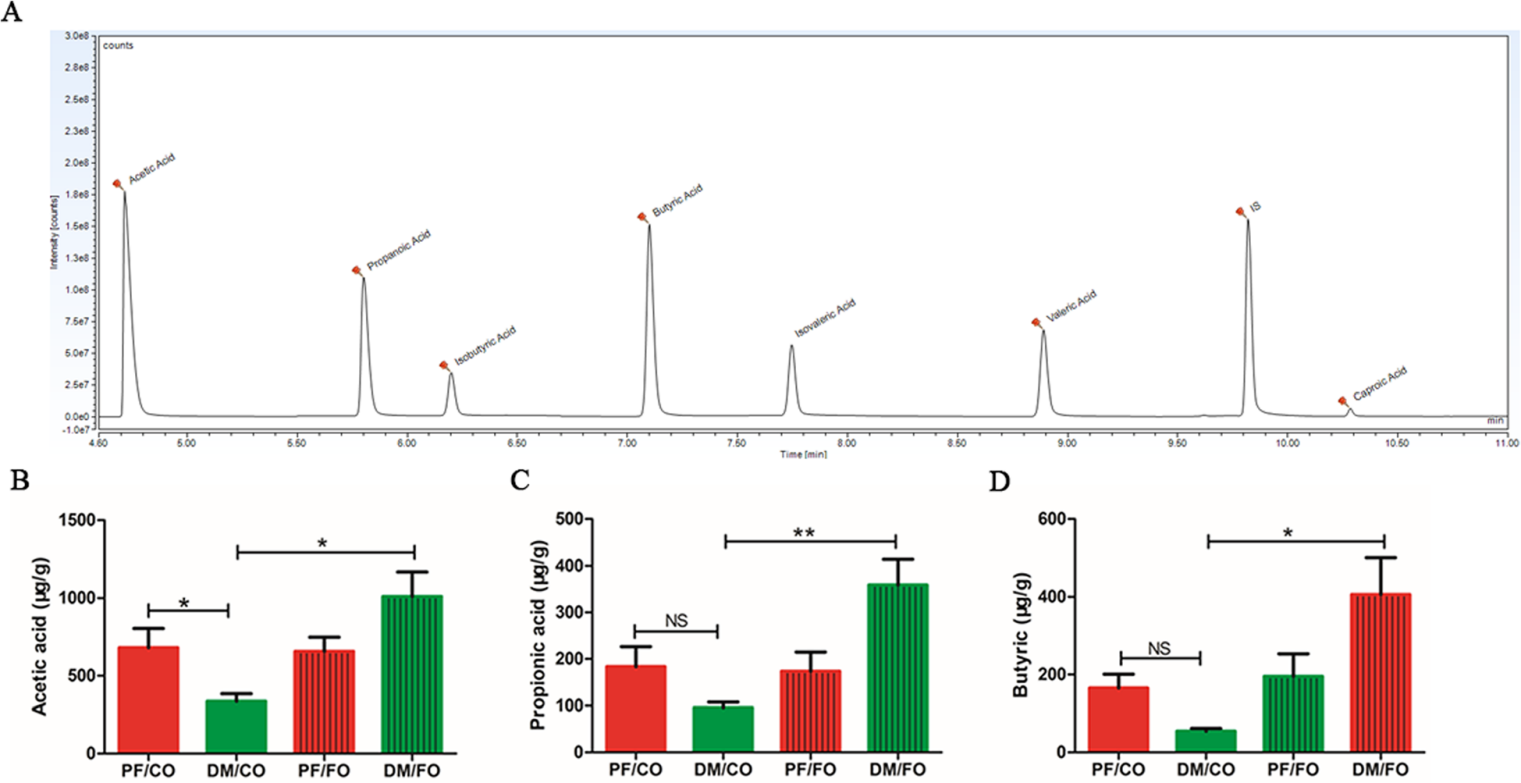
FO increased the content of SCFAs in fecal intestinal metabolites. **a** Sample chromatogram of rats stool. **b** Acetic acid. **c** Propionic acid. **d** Butyric acid (μg/g) (*n* = 5). * *P* < 0.05, ** *P* < 0.001, NS not significant

**Fig. 12:**
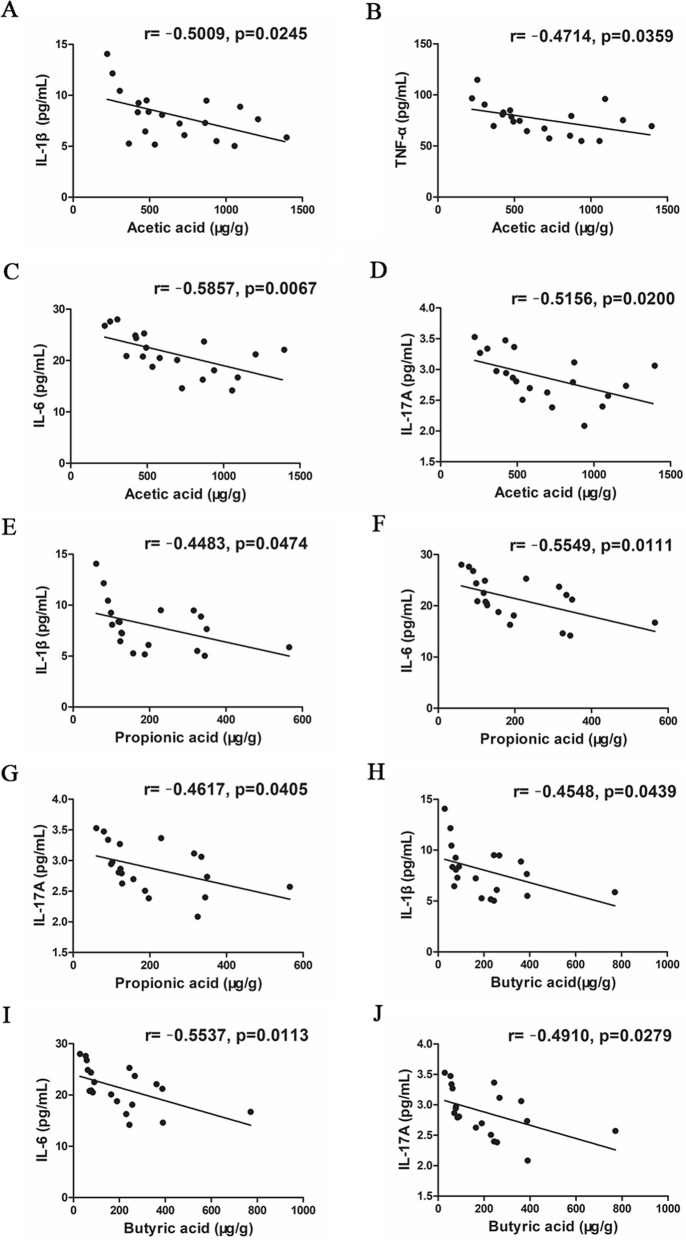
Correlation analysis between SCFAs and liver inflammatory cytokines. **a** Acetic acid and IL-1β; **b** Acetic acid and TNF-α; **c** Acetic acid and IL-6; **d** Acetic acid and IL-17A; **e** Propionic acid and IL-1β; **f** Propionic acid and IL-6; **g** Propionic acid and IL-17A; **h** Butyric acid and IL-1β; **i** Butyric acid and IL-6; **j** Butyric acid and IL-17A

The fecal concentrations of SCFAs mainly including acetic acid, propionic acid and butyric acid, were determined by GC-MS. The results showed that the amount of acetic acid (*P* = 0.046, Fig. 11b), propionic acid (*P* = 0.149, Fig. 11c) and butyric acid (*P* = 0.201, Fig. 11d) of the DM/CO group was decreased compared with that of the PF/CO group. However, the fecal concentration of acetic acid (*P*<0.001, Fig. 11B), propionic acid (*P*<0.001, Fig. 11c) and butyric acid (*P*<0.05, Fig. 11d) in DM/FO group was significantly increased compared with that of the DM/CO group. As shown in Fig. 12, we also found that SCFAs (acetic acid, propionic acid and butyric acid) was negatively correlated with inflammatory cytokines. These results indicated that FO promotes SCFAs production after increasing the abundance of beneficial bacteria.

## Discussion

In this study, we investigated the therapeutic effects and associated mechanisms of dietary FO administration on T2DM induced by STZ and NA in rats. Intraperitoneal injection of STZ is mainly attributed to overproduction of reactive oxygen species (ROS) leading to toxicity of pancreatic cells [23], which regulate the synthesis and release of insulin via the glucose transporter (GLUT) and alkylation of DNA. In contrast, NA prevents excess damage to pancreatic β cells during the induction of T2DM. This T2DM rat model has been widely used to assess the effects and mechanisms of intervention [19, 24–26].

Our results demonstrated that supplementary FO showed more effective in alleviation of T2DM, which may be due to the suppression of inflammation and restoration of gut dysbiosis, potentially providing a theoretical foundation as an inexpensive intervention for prevention and treatment of T2DM.

Consumption of ω-3 PUFAs are beneficial for chronic metabolic diseases including T2DM via anti-inflammation and suppressing oxidative stress in clinical and experimental studies [12, 13]. FO, as a source of a plant-derived ω-3 PUFAs (rich in ALA), has been indicated to show anti-inflammatory properties extensively investigated on the whole body [12, 13]. Wang F et al [27] showed that Perilla oil (rich in ALA) supplementation improved hypertriglyceridemia and gut dysbiosis in diabetic KKAy mice. A randomized double-blind placebo-controlled clinical trial revealed that FO supplementation significantly improved levels of gene expression related to insulin, lipid and inflammation in diabetic patients with coronary heart disease [28]. Our results demonstrated that BM were lower in DM/CO group, but elevated after FO intervention in the STZ-NA induced diabetic rat model, which was consistent with previous study [8], indicating that FO may positively affect nutrients absorption and efficiency of calorie utilization in gastrointestinal tract in T2DM. Yang, Z. H et al [29] found that an increase in ω-3 PUFA levels and the concomitant decrease in the ω-6/ω-3 PUFA level ratio were likely to be involved in the beneficial changes to the metabolic indicators. Interestingly, we also found that FO treatment effectively alleviated routine parameters including FBG, GHb, liver HE (Additional file 5: Figure S4) and metabolic indicators (TC, TG, LDL and HDL), which revealed that FO can regulate the metabolic disorders in consistent with a recent studies [8].

Oxidative stress is considered to be one of the chief factors responsible for the development and progression of T2DM. Haliga, R et al [30] showed that dietary flaxseed reduced renal oxidative stress by improving activity of SOD and decreasing thiobarbituric acid reacting substances (TBARS) in STZ-induced diabetic hamster model. In this study, dietary FO induced an increase of serum oxidative indicator SOD as well as a decrease of MDA, which was paralleled with previous study [31]. Collectively, it is indicated that oxidative stress may be linked to the etiology of T2DM and ameliorated by a healthy diet.

LPS derived from pathogenic bacteria, a causal link between gut microbiota and systemic low-grade inflammation, translocates to liver and binds to TLR-4 to induce inflammatory cascade reaction to ultimately lead to diabetes mellitus [32]. Our data showed that plasma LPS was notably decreased after FO intervention, indirectly demonstrating that integrity and permeability of intestinal mucosa barriers were improved to alleviate peripheral endotoxemia. The damage of intestinal tight junction proteins like Zo-1 and Occludin is essential and critical for LPS translocation. However, the direct evidence of gut permeability and integrity still needs further research to measure and assess the efforts of the intestinal mucosal barrier, including physical tight junctions (Zo-1, Occludin) and other components of the gut barriers involving intestinal mucosal immunity (γδ T cells, intraepithelial lymphocytes (IELs), Tregs, natural killer T (NKT) cells).

Diabetes present a chronic metabolic disease with a low-grade inflammation [33]. Studies have demonstrated that activation of neutrophils and macrophage results to oxidative stress as well as inflammatory cytokines release including IL-1β, IL-6 and TNF-α, leading to the decrease of insulin secretion, hepatic steatosis and a series of metabolic disorders in diabetes [34]. Hotamisligil, G *et.al* [35] firstly showed that the proinflammatory cytokine TNF-α was able to induce insulin resistance.

Accelerating studies have demonstrated that ω-3 fatty acids are considered to possess anti-inflammatory ability in chronic metabolic diseases [13]. In T2DM, inflammation and glucose are interrelated, with reciprocal causation [36]. High glucose concentration leads to process of protein glycation and the production of advanced glycose end products (AGEs). As a consequence, the accumulation of AGEs activates NF-κB to induce TNF-α expression which lead to chronic inflammation [37]. Intakes of dietary ω-3 fatty acids are associated with reduced lymphocyte proliferation and Th1 cell development [38], lower circulating levels of leptin, C-reactive protein and other pro-inflammatory cytokines, as well as a lower risk of infection [39]. It has been shown that supplementation of FO for 8 weeks caused a significant decrease in the levels of glucose tolerance, IL-1β and TNF-α in obese and diabetic [8]. Our results showed that a significant augmentation in a series of pro-inflammatory cytokines including IL-1β, TNF-α, IL-6 and IL-17A in STZ-NA induced diabetic rats. However, the levels of those pro-inflammatory cytokines were dramatically reduced after FO treatment, which was in agreement with a recent study where dietary FO modulated expression of inflammatory genes with alleviation of protein glycation status in STZ-NA induced diabetic rats [40]. The possible mechanism of anti-inflammation is that ALA in FO diet-mediated GPR120 activation and β-arrestin 2 recruitment in the inhibition of TLR4 and TNF-α downstream signaling. But the exact evidence needs to be further researched. IL-10 is an anti-inflammatory cytokine released by Kupffer cells and monocytes. But in present study, we found IL-10 showed no significant difference among diverse groups, which was not paralleled with previous study of T2DM [41]. We speculated that IL-10 maybe play a complicated role in regulating pro-and anti-inflammation during T2DM. Additionally, regulatory immune cells especially regulatory T lymphocytes (Tregs), which may play a critical role in regulation of inflammation to keep maintain immune balance in T2DM. However, the roles of IL-10 and Treg cells in underlying mechanisms of FO-treated T2DM need to be further investigated.

Growing evidences have demonstrated the gut microbiota play a critical role in the development of T2DM [3, 5, 27]. Gut dysbiosis can facilitate LPS entry into systemic circulation through increasing gut permeability, which leads to inflammation and metabolic dysfunction [42]. In this study, at phylum level, we found that *Bacteriodetes* and *Firmicutes* were the most dominant in all four groups, which was similar to the human intestinal flora [43]. *Firmicutes* played a major role in absorbing calories from the diet and storing fat in gut cells. In present study, a decrease of *Firmicutes* after FO treatment revealed that FO may attenuate diabetes via reducing *Firmicutes*-involved in energy absorption. In addition, the ratio of *Firmicutes*-*Bacteroidetes* was increased in diabetic group, suggesting a characteristics of micro-ecological disorder in intestinal microbes [5]. Importantly, dietary FO intervention restored this ratio, demonstrating that dietary FO positively shaping the host microbial ecosystem [44].

In our results, positive correlations of *Firmicutes* with pro-inflammatory IL-1β, TNF-α, IL-6, IL-17A and LPS demonstrated that both pro-inflammatory indicators and *Firmicutes* contributed to pathogenesis of diabetes. In contrast, negative correlations of *Bacteroidetes* with pro-inflammatory indicators may stand for a protection in diabetes. However, the details in relationship between *Firmicutes*-*Bacteroidetes* and inflammation need to be further investigated.

At the genus level, *Blautia* is a gram-positive, anaerobe bacterium belonging to the family *Lachnospiraceae*, which was thought to take part in the development of glucose metabolism disturbances [45]. A recent study also showed that *Blautia*, especially *Blautiacoccoides*, may activate secretion of TNF-α concentration with an even greater extent than the LPS [46]. In our study, we found that increased *Blautia* was positively correlated with inflammatory indicators (IL-1β, TNF-α, IL-6 and LPS) in diabetes. Meanwhile, we found that the abnormal elevated *Blautia* in diabetes was restored by FO intervention. Similarly, ALA rich in Perilla oil supplementation resulted in an decreased abundance of *Blautia* in diabetic KKAy mice [27]. In addition, we found that FO treatment significantly regulated the abundance of *Alistipes* which belongs to family *Rikenellaceae*. Consistently, *Alistipes* was decreased in gestational diabetes mellitus based on a recent study [47]. Furthermore, we found that *Alistipes was* negatively correlated with TNF-α and LPS, demonstrating that *Alistipes* may suppress diabetic inflammation. The above findings suggested that *Alistipes* may be involved in the improvement of diabetes.

More and more studies have suggested that SCFAs, one of critical gut microbiota metabolites, as a link between microbiota and host homeostasis, play an important role in regulation of inflammation, glucose and lipid metabolism. Acetic acid, propionic acid and butyric acid mainly account for 90–95% of the total SCFAs [48]. Acetate and propionate are the main products of *Bacteroidetes* and butyrate is mainly produced by *Firmicutes* [49]. A study showed that ω-3 PUFA exerted a positive action by reverting microbiota-derived SCFAs [50]. Thus, in this study, restores of abnormal decreased acetic acid with FO administration revealed that dietary FO may alleviate diabetes via modulating gut microbiota acetic acid. In addition, increased *Bacteroidetes*, decreased *Firmicutes*, and reduced ratio of *Firmicutes*-*Bacteroidetes* after dietary FO treatment demonstrated that *Bacteroidetes* acted the main source of increased acetate. Intriguingly, dietary FO supplementation also significantly increased propionic acid and butyric acid which showed decreased in diabetes group but without significant difference, suggesting that dietary FO possessed the ability to increase SCFAs within or without diabetes. But a study reported that oral butyrate administration significantly alleviated diabetic-endotoxemia with improved gut integrity and decreased ratio *Firmicutes*-*Bacteroidetes* [51]. The exact role of increased propionic acid and butyric acid after dietary FO intervention needs to be further investigated. The above suggested that dietary FO may regulate gut microbiota metabolites SCFAs, especially acetate with beneficial effects on diabetes.

## Conclusion

This study highlighted that dietary FO supplementation ameliorated T2DM induced by STZ-NA via anti-inflammation, modulating composition of gut microbiota and gut microbiota metabolite acetate in rat, suggesting that it can potentially serve as inexpensive interventions for the prevention and treatment of diabetes.

## Supplementary information

**Additional file 1: Table S1.** Fatty acid composition (%) of dietary fats contained.

**Additional file 2: Figure S1.** Size distribution was estimated by electrophoresis. (number 6-10is the size distribution in NC/CO group, number 11-15 is the size distribution in NC/FO group, number 21-25 is the size distribution in DM/CO group and number 26-30 is the size distribution in DM/FO group). Correlation analysis of LPS and inflammatory cytokines. (A) IL-1β and LPS; (B) TNF-a and LPS; (C) IL-6 and LPS; (D) IL-17A and LPS.

**Additional file 3: Figure S2.** Correlation analysis of LPS and inflammatory cytokines. (A) IL-1β and LPS; (B) TNF-a and LPS; (C) IL-6 and LPS; (D) IL-17A and LPS. Effects of different dietary oil on liver injury and in T2DM. Representative images of hepatic hematoxylin and eosin (H&E) staining. CV, central vein; NH, normal hepatocyte; DH, degeneration of hepatocytes.

**Additional file 4: Figure S3.** NMDS analysis showing difference in terms of species in fecal samples. (A) PF/CO vs. DM/CO; (B) PF/FO vs. DM/FO; (C) DM/CO vs. DM/FO; (D) PF/CO vs. PF/FO. Size distribution was estimated by electrophoresis. (number 6-10 is the size distribution in NC/CO group, number 11-15 is the size distribution in NC/FO group, number 21-25 is the size distribution in DM/CO group and number 26-30 is the size distribution in DM/FO group).

**Additional file 5: Figure S4.** Effects of different dietary oil on liver injury and in T2DM. Representative images of hepatic hematoxylin and eosin (H&E) staining. CV, central vein; NH, normal hepatocyte; DH, degeneration of hepatocytes. NMDS analysis showing difference in terms of species in fecal samples. (A) PF/CO vs. DM/CO; (B) PF/FO vs. DM/FO; (C) DM/CO vs. DM/FO; (D) PF/CO vs. PF/FO.

## Data Availability

The Additional file used and analysed during the current study are available from the corresponding author on reasonable request.

## Abbreviations

ALA: α-linolenic acid
CO: Corn oil
FO: Flaxseed oil
IL: Interleukin
INS: Insulin
LPS: Lipopolysaccharide
MDA: Malondialdehyde
OTUs: Operational taxonomic units
PUFAs: Polyunsaturated fatty acids
SCFAs: Short chain fatty acids
SOD: Superoxide dismutase
STZ-NA: Streptozotocin-nicotinamide
T2DM: Type 2 diabetes mellitus
TLR-4: Toll-like receptor-4
TNF-α: Tumor necrosis factor-α
