# Supplementary material for: Dietary flaxseed oil rich in omega-3 suppresses severity of type 2 diabetes mellitus via anti-inflammation and modulating gut microbiota in rats
Source: Lipids Health Dis. 2020 Feb 7;19:20. doi: 10.1186/s12944-019-1167-4 (PMC7006389; doi:10.1186/s12944-019-1167-4)
Supplement: Supplementary file 1 — Additional file 1: Table S1. Fatty acid composition (%) of dietary fats contained. [file 12944_2019_1167_MOESM1_ESM.pdf]

Additional file 1: Table S1 Fatty acid composition (%) of dietary fats contained.

Fatty acids		% of total fatty acids	
**Common name**	**Symbol**	**Corn oil**	**Flaxseed oil**
Lauric acid	12:0	0.2	0.2
Myristic acid	14:0	0.9	0.7
Palmitic acid	16:0	16.8	8.6
Palmitoleic acid	16:1 n-7	0.4	0.3
Stearic acid	18:0	4.1	5.4
Oleic acid	18:1 n-9	25.7	20.2
Linoleic acid (LA)	18:2 n-6	51.0	14.3
α-Linolenic acid (ALA)	18:3 n-3	1.0	50.3
SFAs		21.8	14.9
MUFAs		26.1	20.4
Total PUFAs		52.0	64.7
n-6 PUFAs/n-3 PUFAs		50.0	0.3
