# Supplementary material for: Dietary flaxseed oil rich in omega-3 suppresses severity of type 2 diabetes mellitus via anti-inflammation and modulating gut microbiota in rats
Source: Lipids Health Dis. 2020 Feb 7;19:20. doi: 10.1186/s12944-019-1167-4 (PMC7006389; doi:10.1186/s12944-019-1167-4)

Additional file 2: Fig. S1 Size distribution was estimated by electrophoresis. (number 6-10 is the size distribution in NC/CO group, number 11-15 is the size distribution in NC/FO group, number 21-25 is the size distribution in DM/CO group and number 26-30 is the size distribution in DM/FO group).

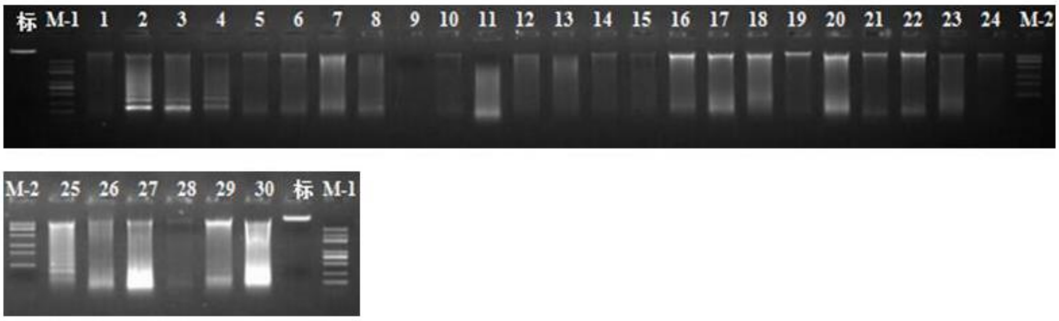

Supplement: Supplementary file 2 — Additional file 2: Figure S1. Size distribution was estimated by electrophoresis. (number 6-10is the size distribution in NC/CO group, number 11-15 is the size distribution in NC/FO group, number 21-25 is the size distribution in DM/CO group and number 26-30 is the size distribution in DM/FO group). Correlation analysis of LPS and inflammatory cytokines. (A) IL-1β and LPS; (B) TNF-a and LPS; (C) IL-6 and LPS; (D) IL-17A and LPS. [file 12944_2019_1167_MOESM2_ESM.pdf]
