# Supplementary material for: Dietary flaxseed oil rich in omega-3 suppresses severity of type 2 diabetes mellitus via anti-inflammation and modulating gut microbiota in rats
Source: Lipids Health Dis. 2020 Feb 7;19:20. doi: 10.1186/s12944-019-1167-4 (PMC7006389; doi:10.1186/s12944-019-1167-4)

Additional file 3: Fig. S2 Correlation analysis of LPS and inflammatory cytokines. (A) IL-1β and LPS; (B) TNF-a and LPS; (C) IL-6 and LPS; (D) IL-17A and LPS.

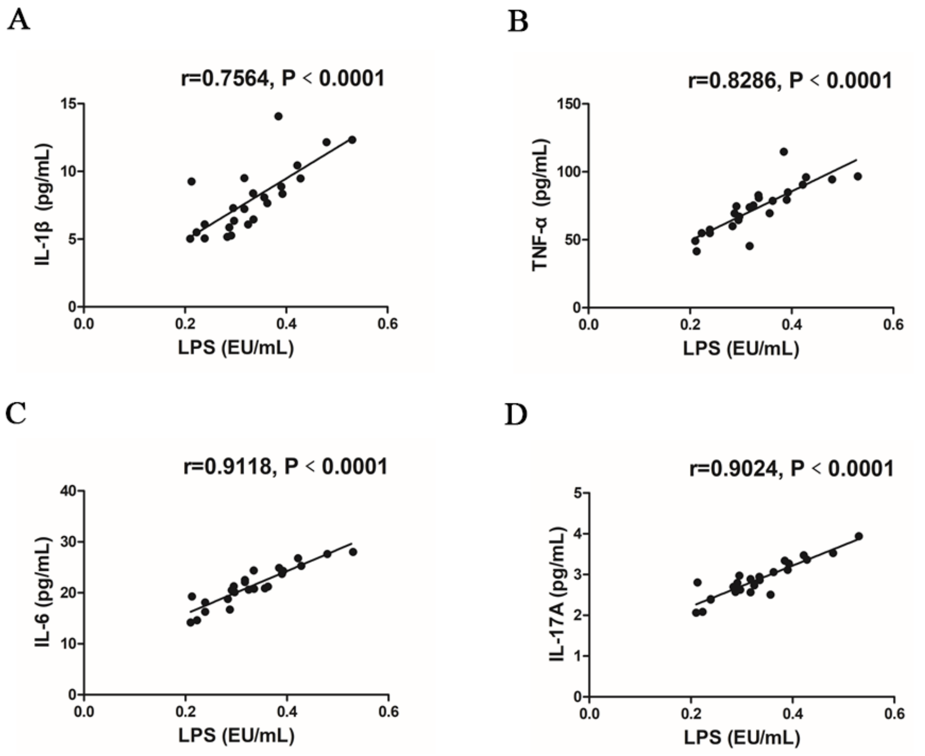

Supplement: Supplementary file 3 — Additional file 3: Figure S2. Correlation analysis of LPS and inflammatory cytokines. (A) IL-1β and LPS; (B) TNF-a and LPS; (C) IL-6 and LPS; (D) IL-17A and LPS. Effects of different dietary oil on liver injury and in T2DM. Representative images of hepatic hematoxylin and eosin (H&E) staining. CV, central vein; NH, normal hepatocyte; DH, degeneration of hepatocytes. [file 12944_2019_1167_MOESM3_ESM.pdf]
