# Supplementary material for: Dietary flaxseed oil rich in omega-3 suppresses severity of type 2 diabetes mellitus via anti-inflammation and modulating gut microbiota in rats
Source: Lipids Health Dis. 2020 Feb 7;19:20. doi: 10.1186/s12944-019-1167-4 (PMC7006389; doi:10.1186/s12944-019-1167-4)

Additional file 4: Fig. S3 NMDS analysis showing difference in terms of species in fecal samples. (A) PF/CO vs. DM/CO; (B) PF/FO vs. DM/FO; (C) DM/CO vs. DM/FO; (D) PF/CO vs. PF/FO.

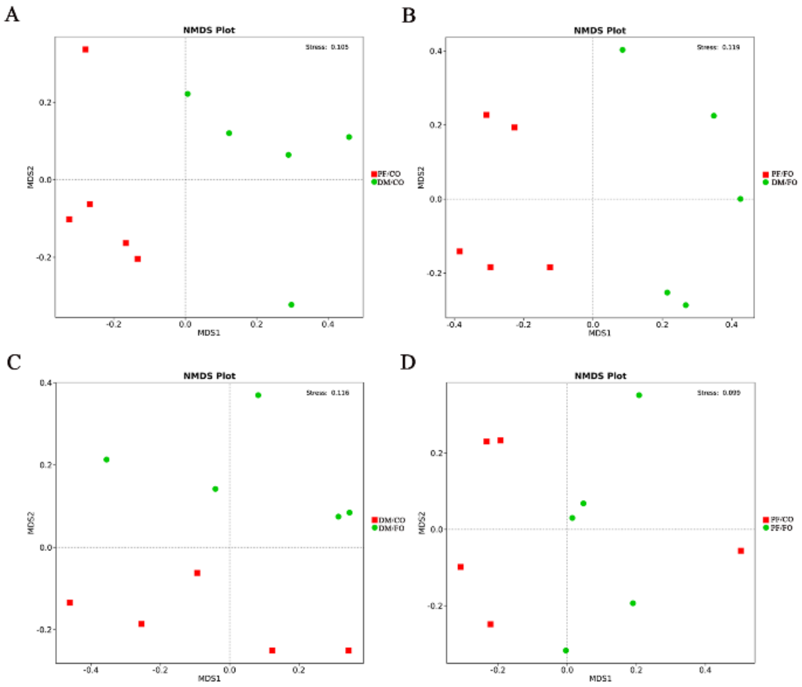

Supplement: Supplementary file 4 — Additional file 4: Figure S3. NMDS analysis showing difference in terms of species in fecal samples. (A) PF/CO vs. DM/CO; (B) PF/FO vs. DM/FO; (C) DM/CO vs. DM/FO; (D) PF/CO vs. PF/FO. Size distribution was estimated by electrophoresis. (number 6-10 is the size distribution in NC/CO group, number 11-15 is the size distribution in NC/FO group, number 21-25 is the size distribution in DM/CO group and number 26-30 is the size distribution in DM/FO group). [file 12944_2019_1167_MOESM4_ESM.pdf]
