# Supplementary material for: Dietary flaxseed oil rich in omega-3 suppresses severity of type 2 diabetes mellitus via anti-inflammation and modulating gut microbiota in rats
Source: Lipids Health Dis. 2020 Feb 7;19:20. doi: 10.1186/s12944-019-1167-4 (PMC7006389; doi:10.1186/s12944-019-1167-4)

Additional file 5: Fig. S4 Effects of different dietary oil on liver injury and in T2DM. Representative images of hepatic hematoxylin and eosin (H&E) staining. CV, central vein; NH, normal hepatocyte; DH, degeneration of hepatocytes.

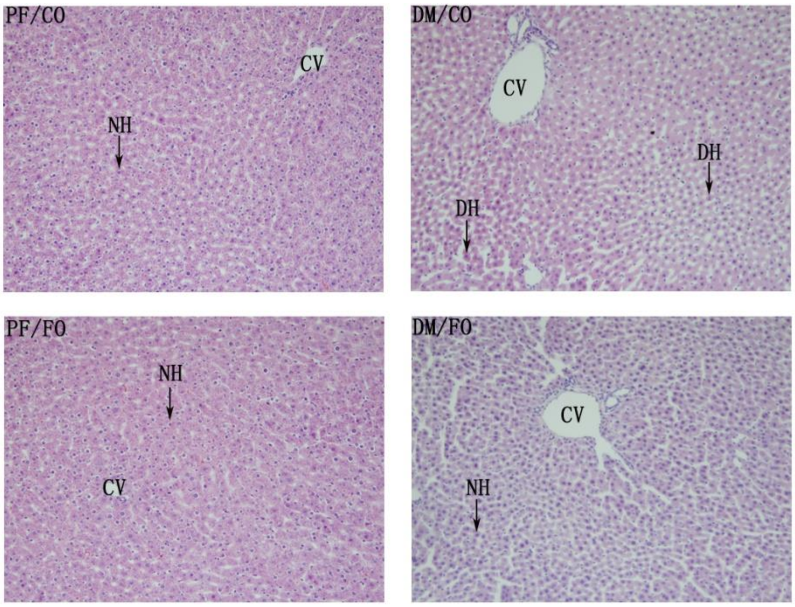

Supplement: Supplementary file 5 — Additional file 5: Figure S4. Effects of different dietary oil on liver injury and in T2DM. Representative images of hepatic hematoxylin and eosin (H&E) staining. CV, central vein; NH, normal hepatocyte; DH, degeneration of hepatocytes. NMDS analysis showing difference in terms of species in fecal samples. (A) PF/CO vs. DM/CO; (B) PF/FO vs. DM/FO; (C) DM/CO vs. DM/FO; (D) PF/CO vs. PF/FO. [file 12944_2019_1167_MOESM5_ESM.pdf]
